# Enhancement of photoluminescence efficiency in GeSe ultrathin slab by thermal treatment and annealing: experiment and first-principles molecular dynamics simulations

**DOI:** 10.1038/s41598-018-36068-x

**Published:** 2018-12-05

**Authors:** Yuliang Mao, Xin Mao, Hongquan Zhao, Nandi Zhang, Xuan Shi, Jianmei Yuan

**Affiliations:** 1 Hunan Key Laboratory for Micro–Nano Energy Materials and Devices, School of Physics and Optoelectronic, Xiangtan University, Hunan, 411105 China; 2 Chongqing institute of Green and Intelligent Technology, Chinese Academy of Sciences, Chongqing, 401120 China; 3 Hunan Key Laboratory for Computation and Simulation in Science and Engineering, School of Mathematics and Computational Science, Xiangtan University, Hunan, 411105 China

## Abstract

The effect of thermal treatment and annealing under different temperatures from 100 °C to 250 °C on the photoluminescence spectroscopy of the GeSe ultrathin slab is reported. After the thermal treatment and annealing under 200 °C, we found that the photoluminescence intensity of A exciton and B exciton in GeSe ultrathin slab is increased to twice as much as that in untreated case, while is increased by ~84% in the photoluminescence intensity of C exciton. Combined by our experimental work and theoretical simulations, our study confirms the significant role of thermal treatments and annealing in reducing surface roughness and removing the Se vacancy to form more compact and smoother regions in GeSe ultrathin slab. Our findings imply that the improved quality of GeSe surface after thermal treatments is an important factor for the photoluminescence enhancement.

## Introduction

In recent years, two-dimensional (2D) semiconductor materials have become the research focus of nanoscience due to their interesting physical properties^1–4^. For example, transition metal sulfide compounds such as MoS_2_ and WSe_2_ have attracted extensive attentions^5–9^. For a few layers 2D materials, van der Waals interactions between the layers are weak. Because of this reason, the electronic structure and physical properties of the 2D materials can be tuned by the reduction of layers^10–12^. However, there are challenge issues about the high resistance, low photoluminescence (PL) quantum efficiency and electron mobility in the study of transition metal sulfide compounds^13,14^. To improve the quantum efficiency of PL^15–17^, high temperature annealing method was used to optimize the structure of ultra-thin MoS_2_ film, which effectively increases the mobility of carriers and reduces the defects on its surface^18^. It was reported that the dependence of annealing temperature and annealing time on the PL intensity of MoS_2_ ultrathin slab^19^. It was found that the PL intensity of MoS_2_ ultrathin slab was increased over 50 times after a 40 min vacuum annealing under 450 °C. It was also found that the PL intensity of WSe_2_ ultrathin slab was increased three times under thermal treatment and annealing^19^. We previously reported that the quantum efficiency of MoS_2_ ultrathin slab under thermal treatments can be increased twice of that under room temperature (RT)^20^.

Recently, the few layer to monolayer germanium selenide (GeSe) was prepared using mechanical stripping and laser thinning method in our group^21^. However, the modulation of quantum efficiency in GeSe ultrathin film by heat treatment and annealing is still unknown^22,23^. Among the group-IV chalcogenides MX (M = Ge, Sn and X = S, Se), GeSe ultrathin film has high electron mobility and behaves light responsiveness^24–26^. The high structural stability of GeSe surface was demonstrated under chemical environment due to its chemical inert surface^27^. Compared to other 2D materials, the direct band gap of GeSe ultra-thin slab overlaps well with the solar spectrum, which plays a key role in the light absorption layer of solar cells and other optoelectronic devices^23,28–30^. Our recent computational work predicted that GeSe monolayer with point defects engineering can be used for gas sensor^31^. Due to active edges of GeSe nanoribbon, our first-principles simulation revealed that its electronic and magnetic properties can be tuned by P atom doping^32^. We also found that the direct band gap of AB-stacking GeSe bilayer can be tuned in a wide energy range under the applied in-plane strains along its armchair direction^33^. As a type of functional material, GeSe ultrathin slab has potential applications in the aspect of photoconductive p-n junction and field effect transistor^34^ and device with giant piezoelectric effects^35^. However, the mechanism of the thermal treatments under different annealing temperature on the PL intensity of GeSe ultrathin slab is still a challenge issue.

In this work, using mechanical stripping and laser thinning technology developed in our group^21^, we firstly prepared the ultrathin GeSe slab on SiO_2_/Si substrate. In order to study the effect of different annealing conditions on the PL efficiency of GeSe ultrathin slab, the samples are annealed in high vacuum and under different temperatures. It was found that after the thermal treatment and annealing under 200 °C, the PL intensity of A exciton and B exciton in GeSe ultrathin slab is increased twice of that under RT, while the PL intensity of C exciton reaches ~84% increasing. Combined by our experimental work and theoretical simulations, our study confirms the significant role of thermal treatments and annealing in reducing surface roughness and removing the Se vacancy to form more compact and smoother regions in GeSe ultrathin slab. Our findings imply that the improvement of the surface smoothness in GeSe samples is an important factor for PL enhancement after thermal treatments.

## Results

### AFM characterization of the laser thinned GeSe nanosheets after annealing at different temperatures

**Figure 1:**
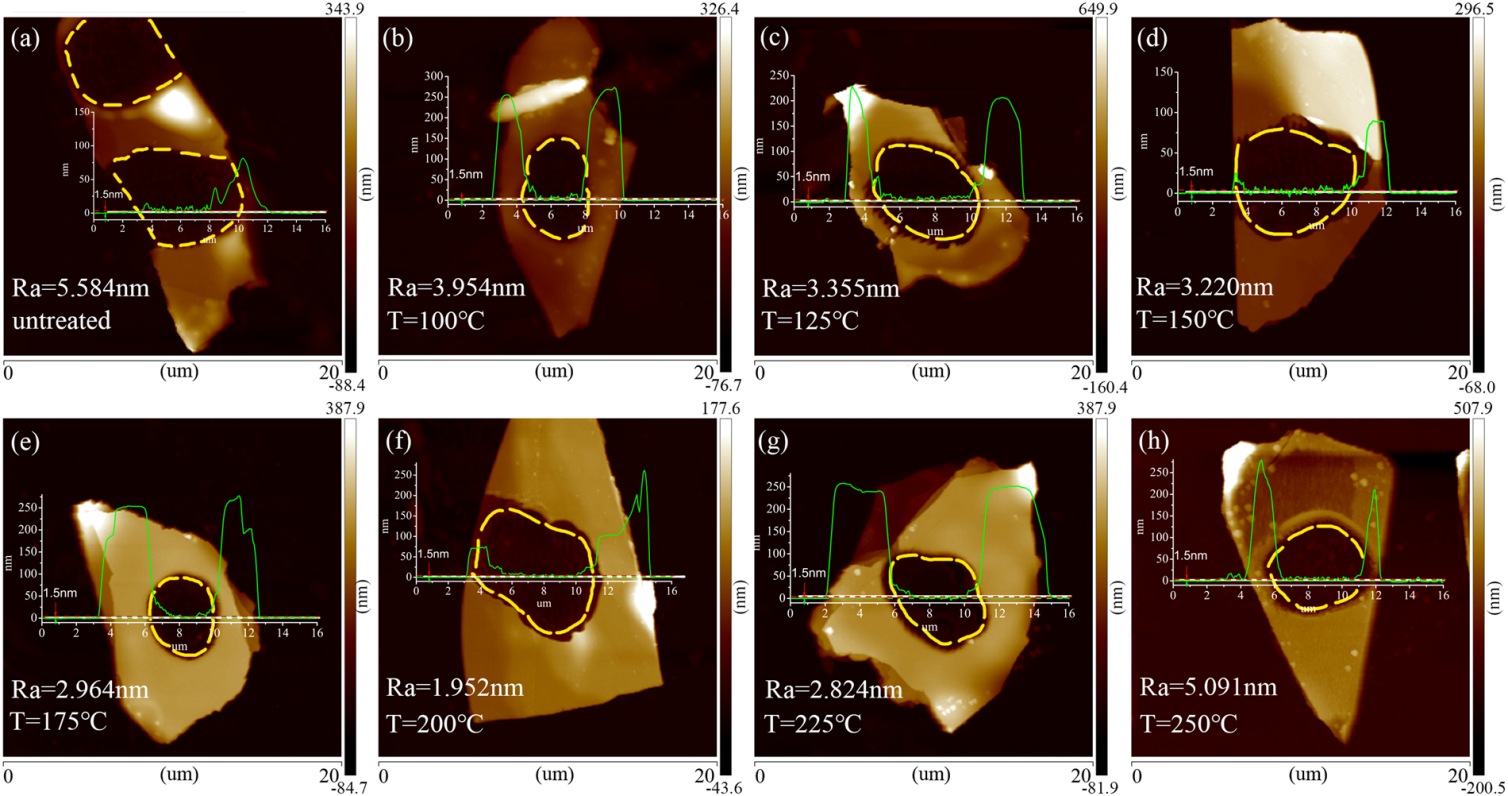
AFM characterization of the laser thinned GeSe nanosheets after annealing at different temperatures. (**a**) AFM image of a thinned GeSe nanosheets without annealing, the annealing temperature is increased step by step from (**b**) 100 °C (**c**) 125 °C (**d**) 150 °C (**e**) 175 °C (**f**) 200 °C (**g**) 225 °C, to (**h**) 250 °C for each sample. Inserted topographic line profiles in the AFM images show the sharpness of the steps between the thinned and pristine zones. The average roughness Ra in laser thinned region is annotated in each image.

The AFM characterization of the laser thinned GeSe nanosheets after annealing at different temperatures is indicated in Fig. 1. In Fig. 1(a–h), the shape and height and the average roughness of a laser thinned GeSe nanosheet are measured through the AFM scan images. The area of the AFM scan for each sample is 20 um × 20 um. The laser power density is 36.8 × 10^4^ W/cm^2^. The sample is continuously irradiated for 5 minutes. Each of the seven samples was treated under inserted topographic line profiles in the AFM images show the sharpness of the steps between the thinned and pristine zones. It can be found from the data that the thickness of each sample after thinning is about 1.5 nm, which shows good uniformity. In order to confirm the effect of thermal annealing on the roughness, we measured the roughness of the thinned GeSe ultrathin slab before and after the annealing at various spots of the samples. Experimental results show that the thermal treatments and annealing have influence on the roughness of the thinned areas under different temperatures of 100 °C, 125 °C, 150 °C, 175 °C, 200 °C, 225 °C and 250 °C, respectively. When temperature is increased from 100 °C to 200 °C, it was found that the average roughness Ra of the thinned surfaces is gradually decreased. As shown in Fig. 1(f), Ra reaches a minimum value of 1.952 nm after the thermal annealing under 200 °C. It means that the smoothness of the GeSe surface of the thinned areas is increased by thermal treatment and annealing under this temperature. When the annealing temperature is higher than 200 °C, it was found that the roughness of the sample surface is increased again.

### SEM characterization of the laser thinned GeSe nanosheets after annealing at different temperatures

**Figure 2:**
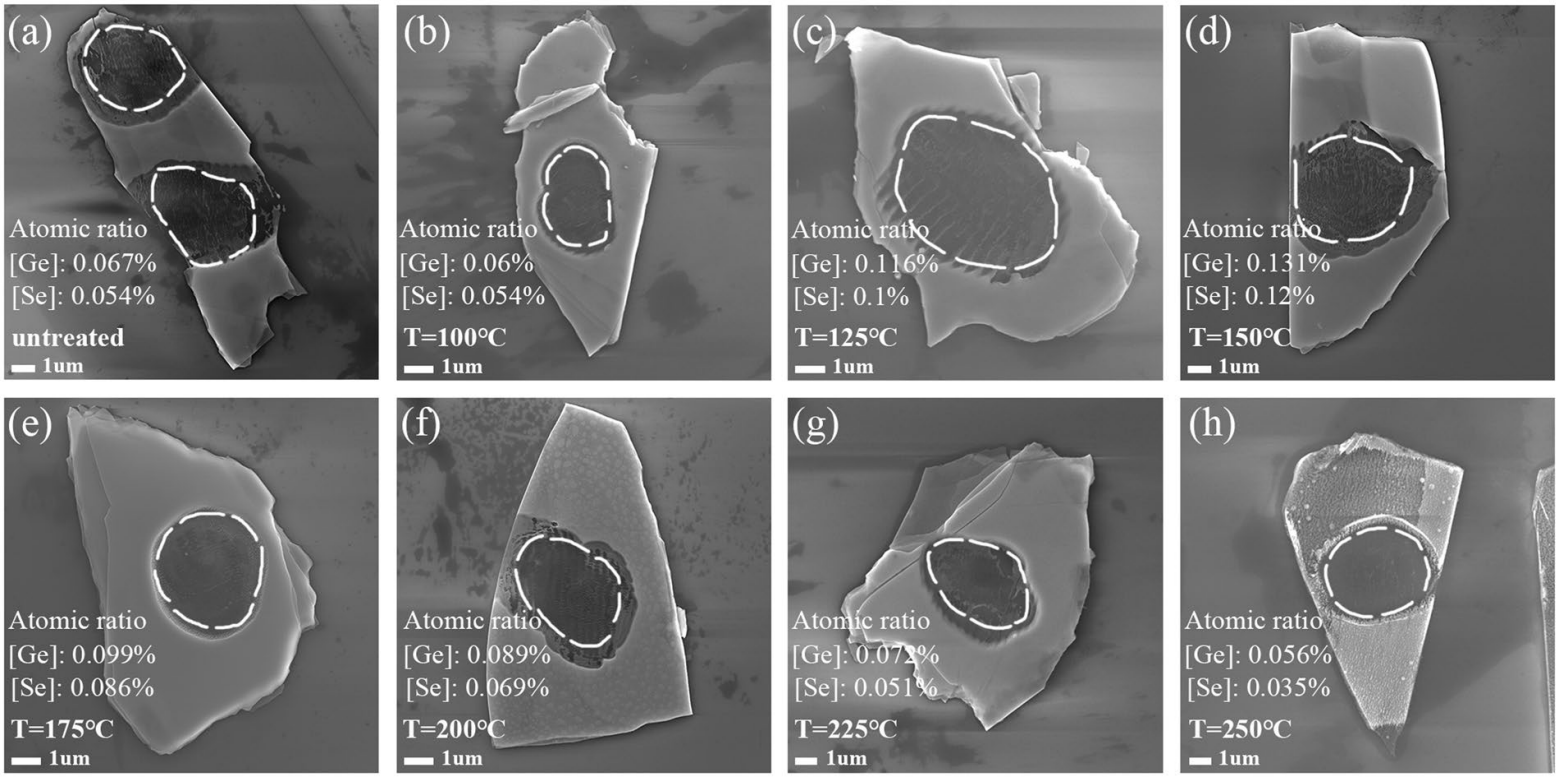
SEM characterization of the laser thinned GeSe nanosheets after annealing at different temperatures. (**a**) SEM image of a thinned GeSe nanosheet without annealing. The annealing temperatures are increased step by step from (**b**) 100 °C (**c**) 125 °C (**d**) 150 °C (**e**) 175 °C (**f**) 200 °C (**g**) 225 °C, to (**h**) 250 °C for each sample, respectively. The element average ratios of Ge and Se atoms of the laser thinned region is annotated in each image.

In order to study the Ge/Se ratio on different spots of the samples after annealing, all samples were characterized by SEM. As shown in Fig. 2, element ratios of Ge and Se are measured by EDS spectrum for each thinned area. The average vacancy rate of Se atom was evaluated by analyzing the proportion of element ratios of Ge and Se in multiple thinned areas. The results show that the Se vacancies are existed in the original thinning area. When the annealing temperature is increased to 150 °C, the ratio of Se vacancies reaches smallest value. Continually increasing the annealing temperature over 150 °C leads to more Se vacancies without the presence of the Se source. The existing of Se vacancies should be responsible for the variation in roughness along with the thermal treatment under different temperatures. However, the segregation and moving of Ge atoms under thermal treatments may also have influence on the roughness of GeSe surface. For this reason, there is a deviation in temperatures between the lowest roughness achieved in 200 °C and the lowest concentration of Se vacancies achieved in 150 °C.

### Annealing and natural cooling temperature dependent PL spectra of GeSe ultrathin slab

**Figure 3:**
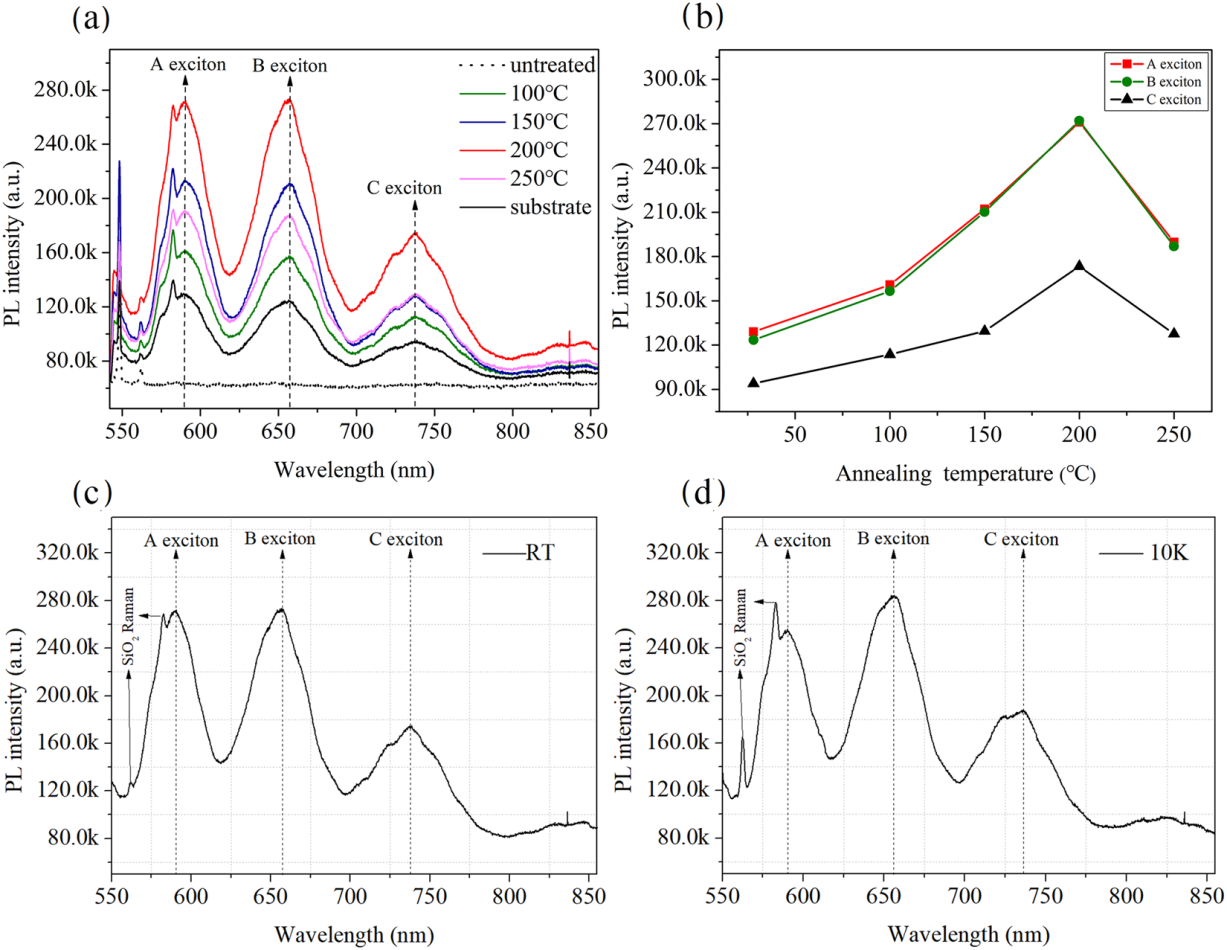
Annealing and natural cooling temperature dependent PL spectra of GeSe ultrathin slab. (**a**) PL spectra of GeSe sheet for various annealing temperatures together with the PL spectrum before thermal annealing. Different colors represent for individual treating temperatures from 100 °C to 250 °C, respectively. (**b**) Treating temperature dependent A exciton, B exciton and C exciton PL peaks intensities; the solid lines are a guide for the eyes. PL spectrum of the 2D GeSe after thermal treatments at 200 °C measured at (**c**) room temperature and (**d**) 10 K.

After the characterization of the ultrathin GeSe sample, we have investigated the annealing temperature dependence of the intensity of PL spectra for A exciton, B exciton and C exciton peaks, respectively. As shown in Fig. 3(a), three peaks centered at 589 nm, 655 nm, and 737 nm are obviously observed. Due to a direct transition at the K point, three positions of peaks are consistent with our previous report^22^. Intensities of these three peaks shows a downward tendency along with the increasing of wavelength. When the treating temperature is increased from 100 °C to 250 °C, the heat annealing significantly changes the intensity of PL spectra. In contrast, the principal PL peak without the treatment of GeSe ultrathin slab (black color) and the spectra of substrate (dotted line) are shown in Fig. 3(a). After annealing under 100 °C, it is found that there is an increasing of 26% both in A exciton peak intensity (I_A_) and B exciton peak intensity (I_B_), respectively, when compared with the case without the treatment of annealing. For C exciton peak intensity (I_C_), the increasing of exciton peak intensity is 21%. Further increasing the annealing temperature leads to more enhancement of exciton peak intensity. For example, if annealing under 150 °C, it results an increasing of ~64% in I_A_ and I_B_, while an increasing of ~38% in I_C_. Under 200 °C, all of the peaks of A exciton, B exciton and C exciton show maximum intensities. As indicated in Fig. 3(b), I_A_ and I_B_ exhibit an increasing more than twice and I_C_ increasing of ~84%, respectively. When the temperature is higher than 200 °C, the intensity of the spectrum is no longer continue to be increased. Similarly, our previous work about the thermal treatments of MoS_2_ thin film under temperatures from 100 °C to 600 °C obtained a maximum value of double PL peak intensity under 200 °C^20^. After 200 °C, it was found that the exciton peak intensity in MoS_2_ sample is decreased linearly. In current study, as shown in Fig. 3(a), the positions of three exciton peaks have no deviation with the variation of temperatures. It implies that the energy gap of GeSe ultrathin slab samples has not been changed. However, from RT to 200 °C, thermal annealing may eliminate interface defects and residual impurities. It leads to a smoother surface of GeSe sample. As the treatment temperature was increased to 250 °C, the uniformity of the sample surface may be destroyed due to the reunion phenomenon of the sample surface. Under this temperature, the intensity of the PL spectral began to decline. Compared with the case in RT as shown in Fig. 3(c), the peak positions of the sample measured under 10 K (Fig. 3(d)) have no obvious change. Moreover, the total intensity of the spectrum and the relative strength of individual peaks are also similar.

### Surface roughness of GeSe monolayer from first-principles molecular dynamics

**Figure 4:**
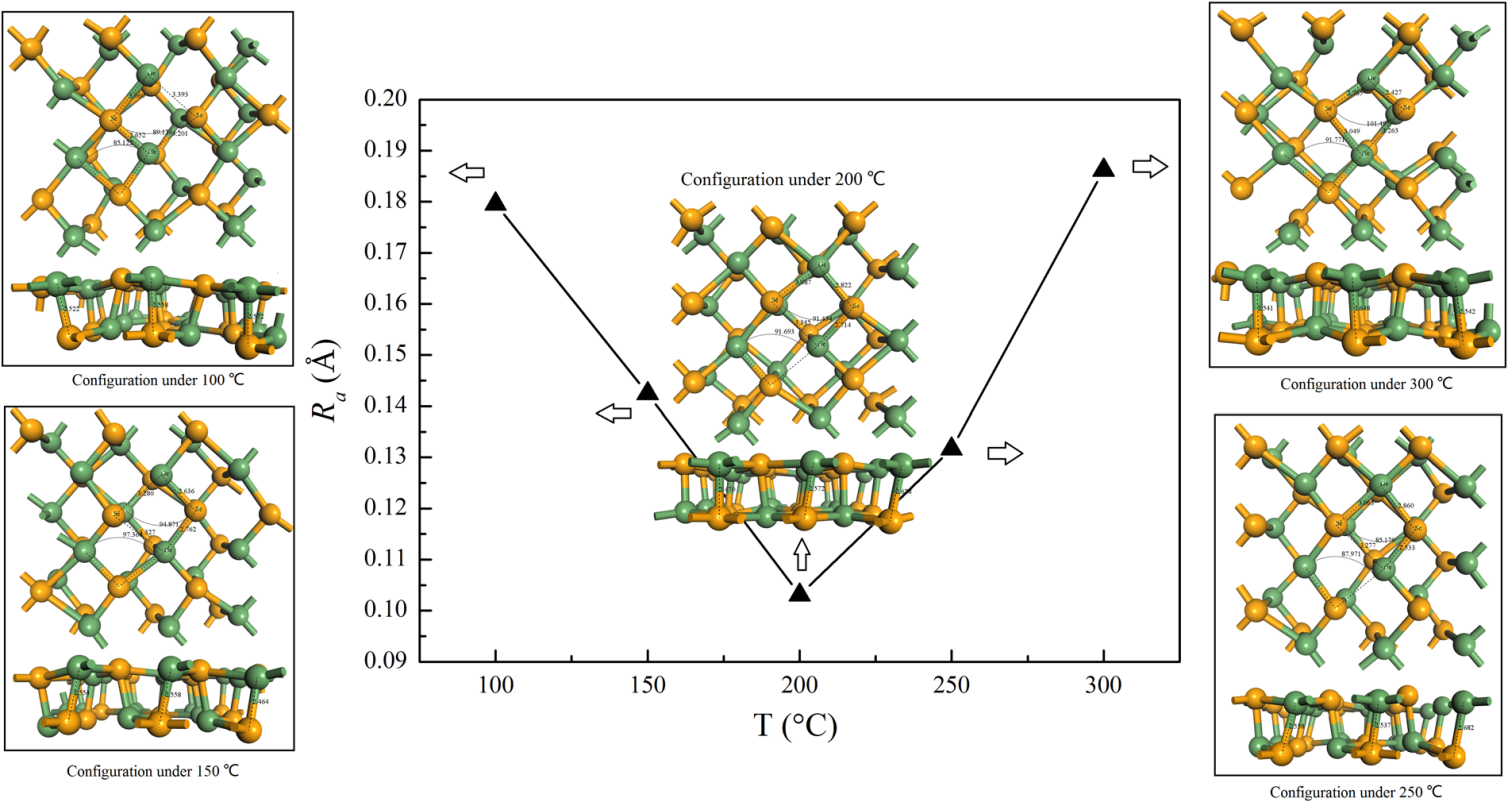
The surface roughness of GeSe monolayer along with the different annealing temperatures, i.e., 100 °C, 150 °C, 200 °C, 250 °C and 300 °C, respectively. The relaxed configurations are also presented, which were obtained from the calculations in a 3 × 3 supercell. In each plot of the configurations, the bond length of some Ge-Se bonds and the bond angles of Ge-Se-Ge or Se-Ge-Se are indicated.

We reported before that the ascension of crystal quality in MoS_2_ can promote the movement of the carrier and direct conversion of quantum efficiency^20^. In quantum wells, it was found that interface roughness over a wide continuous range of well thicknesses has influence on the PL spectroscopy^36^. In this kind of quantum wells, it was reported that the interface roughness with different length scales is responsible for the luminescence features^37^. In theorem, molecular dynamics (MD)^38^ based on density-functional theory (DFT) is an efficient way to study the structural phase transition and material properties of few-layer to monolayer monochalcogenides^39^. In order to investigate the structural roughness of GeSe ultrathin slab under various temperatures, we performed the MD simulations based on DFT. As shown in Fig. 4, MD simulations are used to reveal the correlation between the thermal treatments under different annealing temperatures and the roughness of GeSe monolayer. To quantify the smothness of the surface of GeSe samples, we adopt the following definition of surface roughness in theorem:

$${R}_{a}=\,\sum _{i}\frac{|{Z}_{i}-{Z}_{ave}|}{N}$$

where *Z*_*i*_ and *Z*_*ave*_ represent the exact height and the average height of atoms in the GeSe monolayer, respectively. *N* represents the number of atoms.

In Fig. 4, we presented the surface roughness of GeSe monolayer under different annealing tempertures, i.e., 100 °C, 150 °C, 200 °C, 250 °C and 300 °C, respectively. As indicated in Fig. 4, our simulations indicate that the thermal treatments under different annealing temperatures have great effect on the surface roughness. It’s obvious that the surface roughness of the GeSe monolayer is varied along with the temperatures. Our MD simulations predicte that the roughness has a minimum value under 200 °C. It means that under the temperature of 200 °C, the surface of GeSe samples reaches a very smooth level when compared to the cases under other annealing temperatures. Our simulations illustrated that the variation of surface roughness is related to the annealing temperatures. Until now there is no direct evidence that the annealing temperature has influence on the fluorescence quantum efficiency, whereas the theoritical results proved that the surface roughness is affected by thermal treatments. Our MD results about the roughness of GeSe monolayer imply that the change of roughness in GeSe surface is an important factor for PL enhancement after thermal treatments. The surface roughness obtained from AFM expriments and the consistent simulated results on GeSe monolayer illustrate a main character in the aspect of the quality of the GeSe ultrathin slab samples after thermal treatments and annealing. Our simulations reveal that thermal treatment under 200 °C can increase the smoothness of the surface of ultrathin GeSe slab, which is helpful to increase the fluorescence quantum efficiency in GeSe sample. In another aspect, the samples can produce vacancies with the residual oxygen when they were annealed in vacuum. After that, the vacancies can be combined with charged exciton or free electrons forming a neutral exciton localization. The localization of neutral exciton is very stable and there will be no nonradiative recombination^40–42^, which conduct to the significant enhancement in the spectral intensity of our prepared ultrathin GeSe slab sample.

### The data of roughness and the rate of Se vacancies obtained from experimental samples

**Figure 5:**
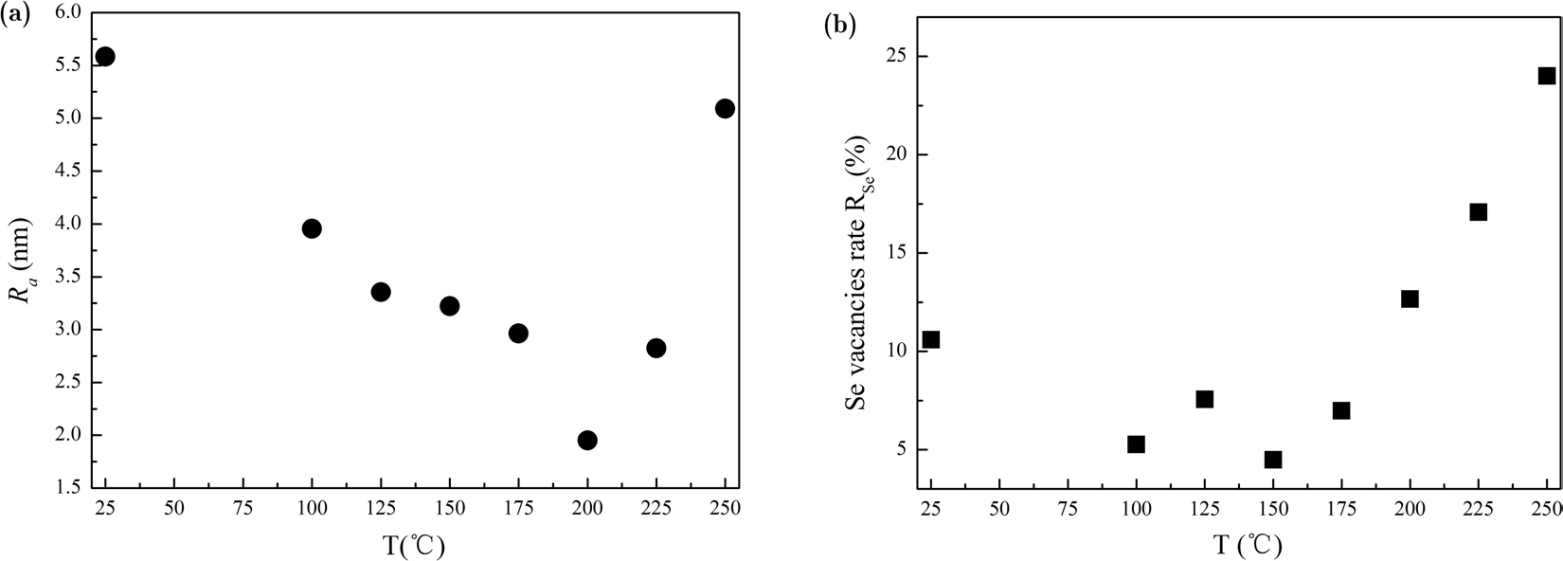
(**a**) The roughness of GeSe samples under room temperature and after thermal treatment under different annealing temperatures obtained from AFM experiments. (**b**) The rate of Se vacancies in GeSe samples under room temperature and after thermal treatment under different annealing temperatures obtained from SEM experiments.

Due to the coexistence of fewlayers and monolayer in prepared GeSe ultrathin slab, the roughness obtained from AFM experiments is an average value. In Fig. 5(a), we replotted the roughness obtained directly from AFM experiments. It clearly shows that the variation of surface roughness in GeSe ultrathin slab samples is related to the annealing temperatures. The lowest roughness obtained from experiment is under the typical annealing temperature of 200 °C, which is the same temperature corresponding to the highest PL intensity. This is a direct evidence that the thermal treatment and annealing has influence on the surface roughness. In Fig. 5(b), we show the rate of Se vacancies obtained from SEM experiments under different annealing temperatures. The definition of Se vacancies rate R_Se_ here can be found in Methods part. It implies that thermal treatment has influence on the rate of Se vacancies. There is a similar trend in the curve with that in the plot of roughness in GeSe samples shown in Fig. 5(a). However, the smallest rate of Se vacancies is appeared under 150 °C. It implies that except the contribution from Se vacancies, other influence on roughness such as the segregations of Ge atoms under thermal treatment is existed. Under thermal treatment and annealing, the Se vacancies can be combined with charged exciton or free electrons forming a neutral exciton localization.

### Local morphologies of Se vacancy in GeSe monolayer from MD simulations

In theoretical aspect, the local morphologies induced by Se vacancy in GeSe monolayer under different annealing temperatures are investigated. The results were obtained from MD simulations under different annealing temperatures corresponding to PL experiments (i.e. 100 °C, 150 °C, 200 °C, and 250 °C, respectively).

**Figure 6:**
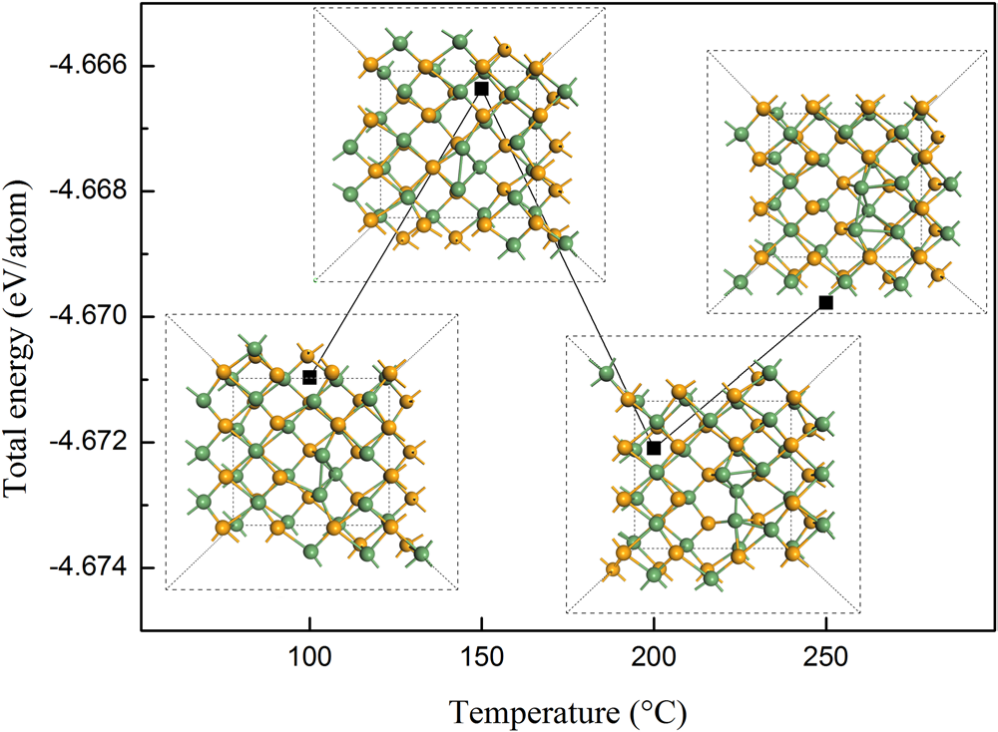
Total energy from MD simulation under different annealing temperatures. The inset plots around each energy point indicate the relaxed configurations after annealing under 100 °C, 150 °C, 200 °C, and 250 °C, respectively. In each configuration, a Se vacancy is constructed in the initio structure of GeSe monolayer. The dashed lines in the insets indicate the used lattice within a 4 × 4 supercell.

As shown in Fig. 6, our results indicate that the total energy for each atom in the relaxed configurations has a fluctuation under different annealing temperatures. Furthermore, our simulation also predicts that the Se vacancy configuration obtained from the annealing of 200 °C reaches the lowest total energy among the four simulated configurations containing a vacancy in each supercell and annealing under corresponding experimental temperatures. The segregation and moving of Ge atom to the position of Se vacancy can be found clearly. It results in the elimination of interface defects induced by the segregation and moving of Ge atom to the position of Se vacancy. As a result, a cage-like structure around the Se vacancy is formed in the configuration after annealing under 200 °C. This cage-like structure around the Se vacancy is preferred in energy to form a compact configuration after annealing. Our simulation implies that the segregation and moving of Ge atom to the site of Se vacancy is helpful to decrease the surface roughness. In other words, it improves the smoothness of GeSe surface.

## Discussion

In the literature, it was reported before that thermal treatment and annealing is an effective way to enhance the mobility and ON/OFF ratio in single-layer MoS_2_ device^43^. In thin quantum wells, previous study^37^ revealed that PL spectra and their temperature dependences can be interpreted in terms of excition localization due to interface roughness with different length scales. In MoSe_2_ monolayer, recent study reported that thermal treatment and annealing significantly improves sample quality by removing certain contaminants and dopants from the surface, resulting the enhancement of PL peak intensity^44^. In another aspect, we noticed that for ultrathin GeSe samples without thermal treatments, as shown in Fig. S4, the SEM images show very rough surface morphologies under room temperature. However, after the samples are annealed at different temperatures, the increased smoothness of the ultrathin GeSe surface is helpful to the PL enhancement. We note that the ultrathin GeSe slab will be volatilized under the temperature about 280 °C. It implies that the surface conditions are crucial factors for PL enhancement.

Thus it is interesting to note that this difference occurs despite the fact that ultrathin GeSe preparation conditions are identical, the only difference on the PL intensity between the samples is the difference in annealing temperatures. The surface smoothness of the few-layers slab plays an important role in PL spectrum of the samples. Our obtained roughness from AFM experiment is a reflection on surface morphologies after thermal treatments and annealing under different temperatures. The ratio of Se vacancies obtained from SEM is also a factor to validate the changes of the surface morphology. Our study shows that under 200 °C the photoluminescence intensity of A exciton and B exciton in GeSe ultrathin slab is increased to twice as much as that in untreated case, while is increased by ~84% in the photoluminescence intensity of C exciton. It implies that heat treatment is an effective way to tune the fluorescence quantum efficiency in GeSe ultrathin slab samples.

## Methods

### Sample preparation and analysis

By using micromechanical exfoliation and laser thinning method^21^, as shown in Fig. S1 in supplementary material, we firstly prepared the ultrathin GeSe samples on SiO_2_/Si substrate. Then, we put the prepared samples into the tube furnace and then pumped it into a vacuum. The molecular pump is kept in vacuum when the pressure in the tube reaches ∼10 Pa. In order to ensure that the samples of studied GeSe ultrathin slab are not affected during the annealing process, the annealing environment is maintained in vacuum state (∼10^−4^ Pa). We consider different annealing temperature of 100 °C, 125 °C, 150 °C, 175 °C, 200 °C, 225 °C, 250 °C, respectively. Then, the samples are cooled naturally to RT. We found that the area without laser thinning will become more transparent when we processed the sample under 300 °C. After annealing on this temperature, it’s difficult to locate the exact location of the sample. However, we still measured the apparent spectra of the sample through positioning. As a result, we can figure out the samples that just have been thinned and are not disappear. The samples were heated by thermal treatment and annealing under 100 °C, 125 °C, 150 °C, 175 °C, 200 °C, 225 °C, 250 °C, respectively.

### Thermal treatment process

The modulation of Photoluminescence intensity of the GeSe sample is treated by thermal treatments. The ultrathin GeSe samples were firstly prepared under the same experimental conditions as before^21^. For comparison, we firstly detected the photoluminescence spectra of monolayer GeSe in the untreated GeSe sample without thermal treatment. Thereafter, the treating temperature was increased from 100 °C to 250 °C in a 25 °C interval. We also performed a thermal treatment under 300 °C, but the GeSe sample becomes transparent under this temperature. The GeSe sample is firstly placed into a vacuum tube. Then the vacuum tube is pumped into a vacuum state using a mechanical pump and a molecular pump. A high-temperature furnace that can set the temperature is used to heat the vacuum tube to different temperature of 100 °C, 125 °C, 150 °C, 175 °C, 200 °C, 225 °C, 250 °C, respectively. Each sample is kept in the tube under the set temperature for 60 minutes. Then the natural cooling method is used to reduce the temperature of the sample to room temperature.

### Measurement Equipment and PL characterization

The thickness and element analysis of the GeSe samples are characterized by atomic force microscopy (AFM, Bruker, Dimension Icon SPM) and scanning electronic microscopy (SEM, JSM-7800F). PL characterization is performed by a laser confocal scanning microscope system and an Andor spectrograph (Andor iDus 416). For the accuracy of the data, the measurement conditions of fluorescence spectrum for all our samples are same. A multiplier of 40 times is used for our laser scanning confocal microscope. The parameters of the spectrometer are set as the integral number is five times, while the integral time is 30 seconds each time. The light valve size of spectrometer is 300. The laser power density of the excitation light is 0.85 × 10^4^ W/cm^2^.

### MD simulations

For pristine GeSe monolayer, MD^38^ simulations were performed on a (*N, V, T*) ensemble on a periodic 3 × 3 supercell of GeSe containing *N* = 36 atoms (18 Ge atoms and 18 Se atoms, respectively). The simulations of MD are within the framework of density functional theory (DFT)^45^. Projector-augmented-wave (PAW) potentials^46^ are used to simulate the ionic motion, while the generalized gradient approximation (GGA) of the PBE^47^ function is used as the exchange and correlation functional. The separated vacuum space of 20 Å above 2D sheet is used to eliminate the interactions between the neighbor supercells. We use a plane cutoff of 440 eV and a *k*-point mesh of 1 × 1 × 1 for sampling the Brillouin zone during the MD calculations. MD were performed and annealing at the finite temperature T = 100 °C, 150 °C, 200 °C, 250 °C and 300 °C, respectively. All the calculated configurations of GeSe monolayer were converged to equilibrate condition under considered temperatures after 3000 MD time steps. Each MD step corresponds to 1 fs. As shown in Fig. S2 in supplementary material, the total energy has a good convergence in our simulations along with the running time. The simulations for vacancy configurations were performed in an enlarged 4 × 4 supercell, in which a Se atom was removed from initio constructed structures. MD simulations were used to achieve the relaxed configurations after annealing at the temperatures corresponding to PL experiments (i.e. 100 °C, 150 °C, 200 °C, 250 °C, respectively).

### S_e_ vacancies rate

In SEM experiment, we adopt the following definition of Se vacancies rate *R*_*se*_:

$$\,{R}_{Se}=\frac{{E}_{Ge}-{E}_{Se}}{{E}_{Ge}+{E}_{Se}}$$

where *E*_*Ge*_ represents the element ratios of Ge and *E*_*Ge*_ represents the element ratios of Se, respectively.

## Electronic supplementary material

Supplementary Material

## References

[1] Wang QH, Kalantar-Zadeh K, Kis A, Coleman JN, Strano MS (2012). Electronics and optoelectronics of two-dimensional transition metal dichalcogenides. Nat. Nanotechnol..

[2] Zhang YQ, Liang YM, Zhou JX (2014). Recent progress of graphene doping. Acta Chim. Sinica..

[3] Geim AK, Novoselov KS (2007). The rise of graphene. Nat. Mater..

[4] Chhowalla M (2013). The chemistry of two-dimensional layered transition metal dichalcogenide nanosheets. Nat. Chem..

[5] Buscema M, Barkelid M, Zwiller V, van der Zant HS, Steele GA (2013). Large and tunable photothermoelectric effect in single-layer MoS_2_. Nano Lett..

[6] Bertolazzi S, Brivio J, Kis A (2011). Stretching and Breaking of Ultrathin MoS_2_. ACS Nano.

[7] Cooper RC (2013). Publisher’s Note: Nonlinear elastic behavior of two-dimensional molybdenum disulfide. Phys. Rev. B.

[8] Castellanosgomez A (2013). Local strain engineering in atomically thin MoS_2_. Nano Lett..

[9] Wang QH, Kalantar-Zadeh K, Kis A, Coleman JN, Strano MS (2012). Electronics and optoelectronics of two-dimensional transition metal dichalcogenides. Nature Nano..

[10] Kuc A, Zibouch NE, Heine T (2011). Influence of quantum confinement on the electronic structure of the transition metal sulfide TmS_2_. Phys. Rev. B..

[11] Splendiani A (2010). Emerging photoluminescence in monolayer MoS_2_. Nano Lett..

[12] Mak KF, He KL, Shan J, Heinz TF (2010). Atomically Thin MoS2: A New Direct-Gap Semiconductor. Phys. Rev. Lett..

[13] Gan X (2013). Chip-integrated ultrafast graphene photodetector with high responsivity. Nature Photon..

[14] Xia FN, Farmer DB, Lin YM, Avouris P (2010). Graphene Field-Effect Transistors with High On/Off Current Ratio and Large Transport Band Gap at Room Temperature. Nano Lett..

[15] Yang Q, Wang W, Xu S, Wang ZL (2011). Enhancing light emission of ZnO microwire-based diodes by piezo-phototronic effect. Nano Lett..

[16] Feng J, Qian X, Huang CW, Li J (2012). Strain-engineered artificial atom as a broad-spectrum solar energy funnel. Nat. Photon..

[17] Hui YY (2013). Exceptional tunability of band energy in a compressively strained trilayer MoS_2_ sheet. ACS Nano..

[18] Eda G (2011). Photoluminescence from chemically exfoliated MoS_2_. Nano Lett..

[19] Tongay S (2013). Broad-range modulation of light emission in two-dimensional semiconductors by molecular physisorption gating. Nano Lett..

[20] Zhao HQ (2016). Bandgap modulation of MoS_2_ monolayer by thermal annealing and quick cooling. Nanoscale.

[21] Zhao H (2018). Band structure and photoelectric characterization of GeSe monolayers. Adv. Funct. Mater..

[22] Hsueh HC, Vass H, Clark SJ, Ackland GJ, Crain J (1995). High-pressure effects in the layered semiconductor germanium selenide. Phys. Rev. B.

[23] Xue DJ (2012). Anisotropic photoresponse properties of single micrometer-sized GeSe nanosheet. Adv Mater..

[24] Rathor A, Sharma V, Heda NL, Sharma Y, Ahuja BL (2008). Compton profiles and band structure calculations of iv–vi layered compounds GeS and GeSe. Radiat. Phys. Chem..

[25] Antunez PD, Buckley JJ, Brutchey RL (2011). Tin and germanium monochalcogenide iv-vi semiconductor nanocrystals for use in solar cells. Nanoscale.

[26] Xu L, Yang M, Wang SJ, Feng YP (2017). Electronic and optical properties of the monolayer group-iv monochalcogenides MX, (M = Ge, Sn; X = S, Se, Te). Phys. Rev. B.

[27] Mukherjee B (2013). Nir Schottky photodetectors based on individual single-crystalline GeSe nanosheet. ACS Appl. Mater. Inter..

[28] Gomes LC, Carvalho A (2015). Phosphorene analogues: isoelectronic two-dimensional group-iv monochalcogenides with orthorhombic structure. Phys. Rev. B.

[29] Osada M, Sasaki T (2009). Exfoliated oxide nanosheets: new solution to nanoelectronics. J. Mater. Chem..

[30] Talapin DV, Lee JS, Kovalenko MV, Shevchenko EV (2010). Prospects of colloidal nanocrystals for electronic and optoelectronic applications. Chem. Rev..

[31] Mao YL, Long LB, Yuan JM, Zhong JX, Zhao HQ (2018). Toxic gases molecules (NH_3_, SO_2_ and NO_2_) adsorption on GeSe monolayer with point defects engineering. Chem. Phys. Lett..

[32] Mao YL, Guo G, Yuan JM, Zhong JX (2018). Edge-doping effects on the electronic and magnetic properties of zigzag germanium selenide nanoribbon. Appl. Surf. Sci..

[33] Mao YL, Xu CS, Yuan JM, Zhao HQ (2018). Effect of stacking order and in-plane strain on the electronic properties of bilayer GeSe. Phys. Chem. Chem. Phys..

[34] Hillhouse HW, Beard MC (2009). Solar cells from colloidal nanocrystals: fundamentals, materials, devices, and economics. Curr. Opin. Colloid. Interface Sci..

[35] Fei R, Li W, Li J, Yang L (2015). Giant piezoelectricity of monolayer group iv monochalcogenides. Appl. Phys. Lett..

[36] Leosson K, Jensen JR, Langbein W, Hvam JM (2000). Exciton localization and interface roughness in growth-interrupted GaAs/AlAs quantum wells. Phys. Rev. B.

[37] Jahn U (1996). Exciton localization, photoluminescence spectra, and interface roughness in thin quantum wells. Phys. Rev. B.

[38] Kresse, G. & Furthmuller, J. Efficient iterative schemes for ab initio total-energy calculations using a plane-wave basis set. *Phys. Rev. B* **54**, 11169–11186 (1996). 10.1103/physrevb.54.11169 9984901

[39] Mehboudi M (2016). Structural phase transition and material properties of few-layer monochalcogenides. Phys. Rev. Lett..

[40] Hu Y (2015). GeSe monolayer semiconductor with tunable direct band gap and small carrier effective mass. Appl. Phys. Lett..

[41] Mak KF, He K, Shan J, Heinz TF (2012). Control of valley polarization in monolayer MoS_2_ by optical helicity. Nat. Nanotechnol..

[42] Skolnick MS (1987). Observation of a many-body edge singularity in quantum well luminescence spectra. Phys. Rev. Lett..

[43] Yin ZY (2012). Single-layer MoS_2_Phototransistors. ACS Nano.

[44] Rogers C, Gray D, Bogdanowicz N, Mabuchi H (2018). Laser annealing for radiatively broadened MoSe_2_ grown by chemical vapor deposition. Phys. Rev. Mater..

[45] Hohenberg, P. & Kohn, W. Inhomogeneous electron gas. *Phys. Rev. B* **136**, 864–871 (1964).

[46] Blöchl PE (1994). Projector augmented-wave method. Phys. Rev. B.

[47] Perdew JP, Burke K, Ernzerhof M (1996). Generalized gradient approximation made simple. Phys. Rev. Lett..

