# Supplementary material for: Enhancement of photoluminescence efficiency in GeSe ultrathin slab by thermal treatment and annealing: experiment and first-principles molecular dynamics simulations
Source: Sci Rep. 2018 Dec 5;8:17671. doi: 10.1038/s41598-018-36068-x (PMC6281588; doi:10.1038/s41598-018-36068-x)
Supplement: Supplementary file 1 — Supplementary Material [file 41598_2018_36068_MOESM1_ESM.pdf]

Supplementary material

Enhancement of photoluminescence efficiency in GeSe ultrathin slab by thermal treatment and annealing: experiment and first-principles molecular dynamics simulations

Yuliang Mao[a,*], Xin Mao[a,b], Hongquan Zhao[b,*], Nandi Zhang[a], Xuan Shi[b], Jianmei Yuan[c]

[a] Hunan Key Laboratory for Micro–Nano Energy Materials and Devices, School of Physics and Optoelectronic, Xiangtan University, Hunan 411105, China

[b] Chongqing institute of Green and Intelligent Technology, Chinese Academy of Sciences, Chongqing 401120, China

[c] Hunan Key Laboratory for Computation and Simulation in Science and Engineering, School of Mathematics and Computational Science, Xiangtan University, Hunan 411105, China

1. Sample preparation

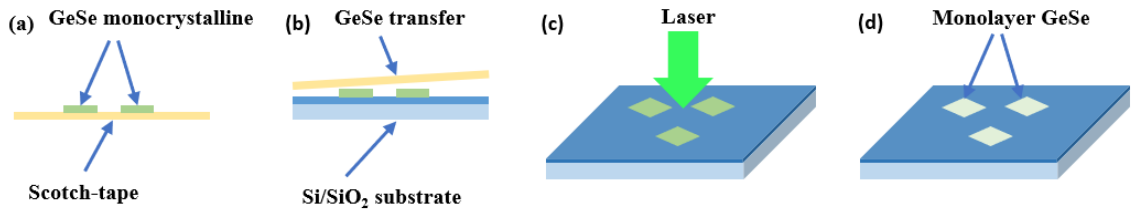

Fig. S1. Schematic illustration of monolayer GeSe preparation. (a) GeSe nanocrystals are fully-picked up by the Scotch-tape. (b) Transfer the GeSe crystals to the surface of Si/SiO_2 substrate. (c) The principle of laser thinning sample. (d) The monolayer of GeSe sample.

In order to study the correlation between the treating temperature and photoluminescence spectra of the single layer GeSe samples, the pristine GeSe nanosheets with thicknesses of 100～200 nm are exfoliated from a commercially available GeSe monocrystalline (2D Semiconductors, Inc. U.S.) by

*Corresponding authors, E-mail: ylmao@xtu.edu.cn; hqzhao@cigit.ac.cn

using the scotch-tape micromechanical cleavage technique method, then GeSe nanosheets are transferred to a Si wafer substrate covered with a 2 um thick amorphous SiO_2 layer. Because of the fragility, it is difficult to use micromechanical exfoliation method to prepare monolayer GeSe samples, therefore we adopt a laser thinning technology to prepare monolayer GeSe samples. The principle of thinning the GeSe sample is to focus the laser on the surface of the sample to reduce the number of sample layers. The vacuum environment is kept during our experiment in order to prevent oxidation from thinning the sample. According to our previous experimental data, the pristine exfoliated GeSe samples are reduced to a single layer by an excitation LPD of 36.7×10^4 W/cm^2 for 5 minutes in vacuum. Our method in the preparation of GeSe samples in current work is same as that in our previous report [S1].

2. Convergence of first-principles molecular dynamics simulations

Motivated by the experimental work at different annealing temperatures, first-principles molecular dynamics (MD) [S2] were performed on a (N, V, T) ensemble on a periodic 3×3 supercell of GeSe containing N=36 atoms (18 Ge atoms and 18 Se atoms, respectively). The simulations of MD are within the framework of density functional theory (DFT) [S3]. Projector-augmented-wave (PAW) potentials [S4] are used to simulate the ionic motion, while the generalized gradient approximation (GGA) of the PBE [S5] function is used as the exchange and correlation functional. The separated vacuum space of 20 Å above 2D sheet is used to eliminate the interactions between the neighbor supercells. We use a plane cutoff of 440 eV and a k-point mesh of 1×1×1 for sampling the Brillouin zone during the MD calculations. MD were performed and annealing at the finite temperature T=100 ℃, 150 ℃, 200 ℃, 250 ℃ and 300℃, respectively . All the calculated configurations of GeSe monolayer were converged to equilibrate condition under considered temperatures after 3000 MD time steps. In each MD step corresponds to 1 fs. As shown in Fig. S2, the total energy has a good convergence in our simulations along with the running time.

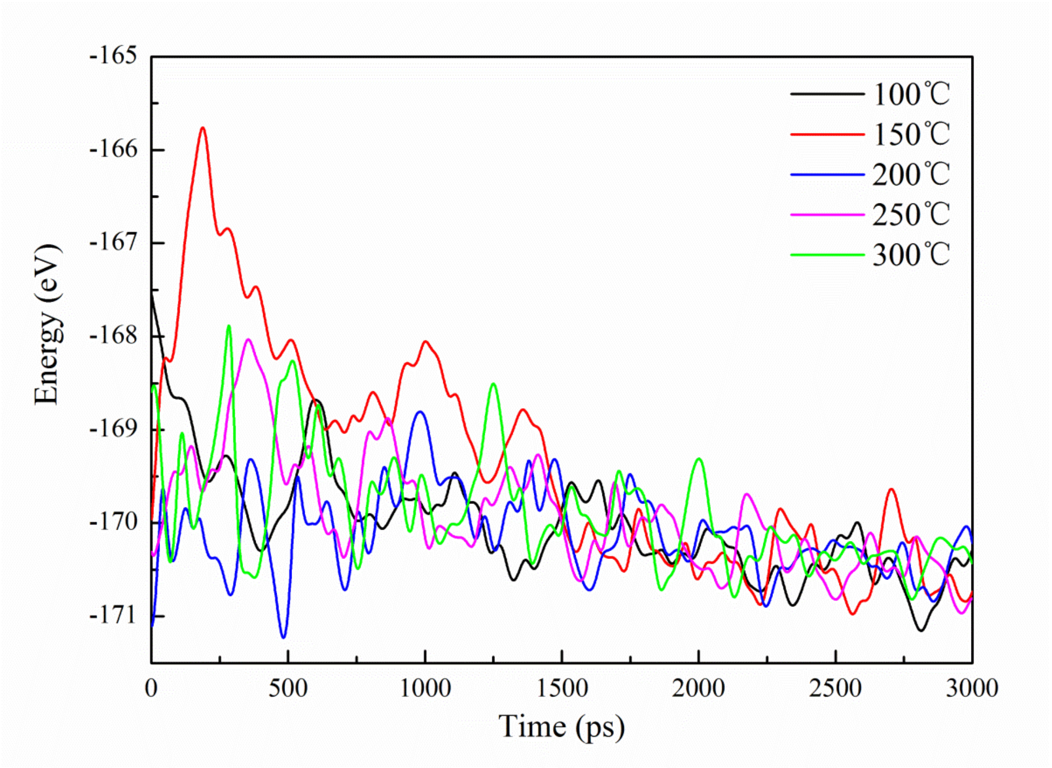

Fig. S2. The convergence of total energy (eV) and running time (ps) under different annealing temperatures.

3. S$_e$ vacancies rate from AFM experiments

Figure S3 [same with Fig. 5(b) in main text] shows the Se vacancies rate of the laser thinned region of 2D GeSe samples along with the different annealing temperatures for 1 hour at 100 ℃, 125 ℃,150 ℃, 175 ℃, 200 ℃, 225 ℃ and 250 ℃, respectively. We adopt the following definition of Se vacancies rate R_{Se}:

$$R_{Se} = \frac{E_{Ge} - E_{Se}}{E_{Ge} + E_{Se}}$$

where E_{Ge} represents the element ratios of Ge and E_{Se} represents the element ratios of Se, respectively.

As shown in Figure S3, the Se vacancies rate of the laser thinned region of GeSe nanosheets after laser thinning is about 10% without thermal treatment. When the treating temperature is increased from 100 °C to 150 °C, the Se vacancies rate changes slightly and slowly decreases. The Se vacancies rate reaches the minimum about 4% after the thermal annealing and annealing under

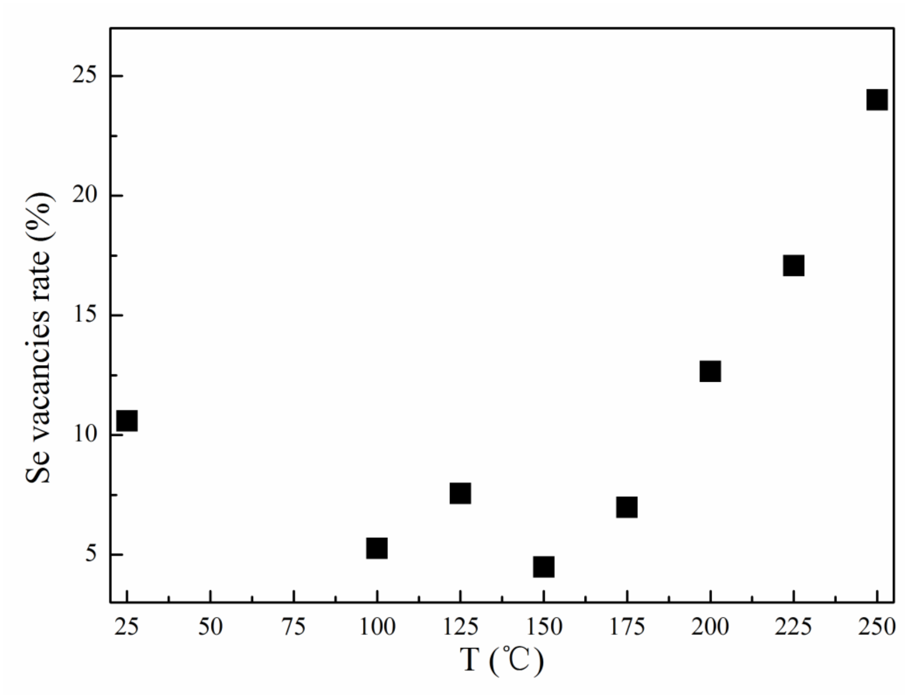

Figure S**3**. The Se vacancies rate of the laser thinned region of GeSe nanosheets along with the different annealing temperatures for 1 hour at 100 ℃, 125 ℃,150 ℃, 175 ℃, 200 ℃, 225 ℃ and 250 ℃, respectively.

150 °C. It's worth noting that the Se vacancies rate is increased greatly as the temperature increased from 100 °C to 150 °C. A maximum Se vacancies rate about 24% of the laser thinned region of GeSe nanosheet under the same measurement conditions was observed at the treating temperature of 250 °C.

4. Surface morphologies of GeSe sample under room temperature

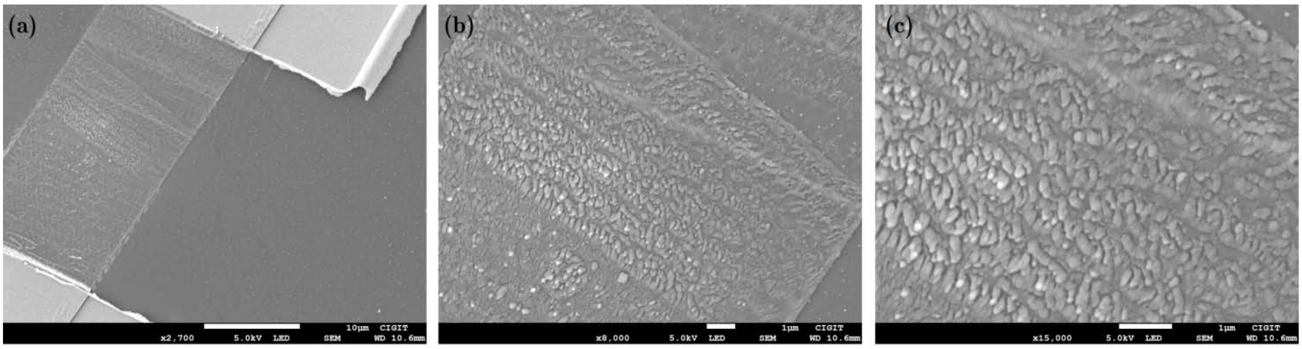

Figure S**4**. Surface morphologies of GeSe ultra-thin slab under room temperature. The magnification corresponding to the original size is (a) 2700 times, (b) 8000 times, and (c) 15000 times, respectively.

In Fig. S4, the scanning electron microscopy (SEM) images for the prepared untreated GeSe ultrathin film are indicated.Under room temperature, these images show very rough surface morphologies of prepared ultrathin GeSe sample.

References

[S1] Zhao, H., Mao, Y., Mao, X., Shi, X., Xu, C., Wang, C., Zhang, S. M. & Zhou, D. H. Band structure and photoelectric characterization of GeSe monolayers. *Adv. Funct. Mater.* **27**, 1704855 (2018).

[S2] Kresse, G., Furthmuller, J. Efficient iterative schemes for ab initio total-energy calculations using a plane-wave basis set. *Phys. Rev. B* **54**, 11169-11186 (1996).

[S3] Hohenberg, P., Kohn, W. Inhomogeneous electron gas. Phys. Rev. B 136, 864-871 (1964).

[S4] Blöchl, P. E., Projector augmented-wave method. *Phys. Rev. B* **50**, 17953 (1994).

[S5] Perdew, J. P., Burke, K., Ernzerhof, M. Generalized gradient approximation made simple. *Phys. Rev. Lett.* **77**, 3865 (1996).
